# The Biocompatibility and Cellular Response of Geristore in Comparison With Different Glass Ionomer Materials on Human Gingival Fibroblast Cells: A Comparative Study on Deep Marginal Elevation

**DOI:** 10.1155/ijbm/4877922

**Published:** 2026-08-23

**Authors:** Mahsa Radafshar, Ardavan Parhizkar

**Affiliations:** ^1^ Department of Endodontics, Faculty of Dentistry, Tehran Medical Sciences, Islamic Azad University, Tehran 194685-3314, Iran, azad.ac.ir; ^2^ Department of Dental Biomaterials, Dental Materials Research Centre, Tehran Medical Sciences Campus, Islamic Azad University, Tehran 194685-3314, Iran, azad.ac.ir; ^3^ Iranian Centre for Endodontic Research, Research Institute for Dental Sciences, Shahid Beheshti University of Medical Sciences, Tehran 198396-3113, Iran, sbmu.ac.ir; ^4^ Department of Endodontics, School of Dentistry, Tehran University of Medical Sciences, Tehran 143995-5991, Iran, tums.ac.ir

**Keywords:** cell adhesion, deep marginal elevation, Geristore, gingival fibroblasts, glass ionomer

## Abstract

**Introduction:**

The aim of the current study was to evaluate the biological response(s) of human gingival fibroblast (HGF) cells to Geristore (GS) and compare them with those of conventional glass ionomers (GIs) to determine their impacts on performance and success in deep margin elevation techniques.

**Methods:**

Samples of GS, GI Fuji II, and resin‐modified glass ionomer (RGI) Fuji II LC were prepared as 6 × 2 mm discs under aseptic conditions and then incubated in 24‐well plates. Next, cytotoxicity was investigated using MTT assay after 48 h of exposure with HGFs. In addition, cell morphology was studied via scanning electron microscopy following 48‐h incubation. Furthermore, cell adhesion was analyzed using fluorescent staining with phalloidin/4′,6‐diamidino‐2‐phenylindole (DAPI).

**Results:**

The MTT assay demonstrated that all tested materials exhibited biocompatibility and nontoxicity, with significantly high cell viability after 48 h (*p* < 0.05). Notably, GS showed superior metabolic activity relative to GI and RGI (*p* < 0.001 and *p* < 0.0001, respectively). Additionally, microscopic analysis revealed that GS promoted the most pronounced fibroblastic morphology and cellular processes. Fluorescent staining confirmed higher cell adhesion density on GS compared to GI and RGI, particularly after 48 h.

**Conclusions:**

The obtained findings could position GS as a promising candidate for scenarios demanding biological compatibility and reparative capabilities in dental applications, specifically in deep margin elevation.

## 1. Introduction

Dental defects present significant clinical hurdles, specifically in terms of access, isolation, management, and adaptation of margins as well as emergence profiles [1]. Consequently, tooth restoration(s) can be a challenge when the crown is shortened due to the damage to occlusal or incisal surfaces or if/when the gingival margin is highly positioned [2]. The aforementioned issues and alike can hinder the ability to (i) achieve adequate resistance and retention, (ii) affect aesthetic outcomes, and (iii) restrict access to caries located subgingivally [3]. Nowadays, dentists frequently encounter decisions regarding teeth having significant subgingival carious lesions or fractures [2]. The potential for restoration is affected by the amount of healthy tooth structure above the bone level and the remaining ferrule on the tooth [4].

Two principal approaches are considered to address the abovementioned issues: crown lengthening (CL) and deep margin elevation (DME) [5]. The CL technique has its constraints, especially when dealing with short roots, as removing bone can create an unfavorable crown–root ratio or risk exposing the furcation area [4]. In contrast, DME involves repositioning subgingival margins to a more coronal position via applying multiple layers of the direct resin‐based composite restorative material to the deep margins, enhancing the overall margin placement [3]. One of the fundamental challenges in DME is the selection of appropriate restorative materials that ensure clinical performance and promote healing/repair of the periodontal tissues [6].

To achieve optimal results in DME, it is essential to use materials that prevent the ingress of bacteria and facilitate the development of a healthy periodontal environment around the restoration [2, 6–8]. In addition, ideal restorative materials for DME should well adhere to the tooth structure, provide a sufficient seal, remain insoluble in tissue fluids, and exhibit dimensional stability [8]. Furthermore, they should be easy to manipulate, demonstrate adequate compressibility, and show biocompatibility with human tissues [6]. Since the restorative materials are often applied in the proximity to living periapical tissues, their compatibility with the mentioned tissues is crucial for achieving a favorable cellular response [7].

Biocompatibility of dental materials is vital for minimizing the inflammatory response and enhancing tissue repair [9]. A biocompatible material should exhibit low toxicity and not provoke significant inflammatory reactions. If a reaction occurs, it should be minimal and transient [10]. Materials can be considered biocompatible if the inflammatory response diminishes to negligible levels within a suitable time frame [11]. The cytotoxicity of restorative materials, particularly when used in procedures like DME, can adversely impact the survival of periodontal cells, potentially leading to adverse outcomes [12]. Therefore, dental materials should ideally promote the repair process or be biologically inert [13].

A variety of materials have been employed in DME and CL, including glass ionomers and resin‐based dental composites [14]. Glass ionomers are principally valued for their versatility and capability to modify properties based on their formulation, making them suitable for various clinical applications [15]. Besides, glass ionomers are often more aesthetically pleasing than traditional metallic restorations and can demonstrate anticariogenic properties owing to incorporated fluorine [13]. The evolution of glass ionomer cements has led to the development of hybrid versions, known as resin‐modified glass ionomer (RGI) cements, which combine the advantages of traditional glass ionomers with improved handling properties associated with light‐cured composites [16].

Geristore (GS), a dual‐cured polyacid‐modified resin glass ionomer, has been proposed as an effective material for DME due to its favorable biological properties [17, 18]. Additionally, GS exhibits low solubility, excellent bonding to the dental structure, a dual‐cure setting mechanism that minimizes curing shrinkage, high fluoride release, and outstanding biocompatibility [19]. Observational studies assessing the impact of different retrograde materials on gingival fibroblasts have shown that GS promotes superior cell attachment and marginal adaptation [9, 20]. Furthermore, the findings of a clinical study have indicated that GS may outperform mineral trioxide aggregate as a restorative option, particularly in the crestal and subcrestal regions. Clinical and radiographic follow‐ups at 1, 3, and 5 years have demonstrated positive and successful outcomes for the stated approach [21].

The necessity for biocompatible materials in DME emphasizes the importance of evaluating their biological properties. Given the limited knowledge on the cytotoxicity of GS in comparison with conventional glass ionomers, the current study aimed to evaluate the biological response(s) of periodontal tissues to GS and its possible implications for DME. Moreover, the presented research investigated the viability, adhesion, and morphological characteristics of HGF cells in response to GS compared to other glass ionomer materials, contributing to a better understanding of GS biocompatibility and enhancing clinical outcomes in restorative dentistry.

## 2. Materials and Methods

### 2.1. Cell Culture

Human gingival fibroblast (HGF)‐1 RT1 cells (HGF‐1 RT1, Pasteur, Tehran, Iran) were sourced from the “National Cell Bank of the Pasteur Institute” in Iran. The cells were cultured in the DMEM (Denazist, Mashhad, Iran), with the addition of 10% fetal bovine serum (Sigma, St. Louis, MO), along with penicillin at a concentration of 100 U/mL (Sigma) and streptomycin at 100 μg/mL (Sigma). Then, the cultures were kept in a humidified incubator with 5% CO_2_ set at 37°C. The culture medium was refreshed every 3‐4 days, and all experiments employed cells from the third passage.

### 2.2. Sample Preparation

The materials of GS (DenMat, USA), GI (Fuji II, GC corporation, Tokyo, Japan), and RGI (Fuji II LC, GC corporation, Tokyo, Japan) were sourced from commercial suppliers. All discs were fabricated with identical dimensions (6 mm in diameter and 2 mm in thickness) under aseptic conditions and prepared strictly according to the manufacturers’ instructions to ensure consistency amongst groups. Standardizing disc size and preparation conditions were intended to minimize variability related to surface area, material volume, and setting characteristics across the tested materials. Glass coverslips were used as an inert, nonbioactive control surface for baseline comparison in all experiments. The use of glass coverslips as a reference control is common in in vitro cytotoxicity and cell–material interaction studies involving fibroblasts, particularly for the normalization of cell viability and adhesion data. Although glass does not represent an optimal substrate for gingival fibroblast attachment, it was intentionally selected as a baseline reference rather than a biologically favorable control, enabling relative comparison of cellular responses across the tested restorative materials [22, 23]. Subsequently, the discs were transferred to 24‐well culture plates, with 3 mL of DMEM added to each well. The plates were then incubated in a 5% CO_2_ and 95% air environment at 37°C.

### 2.3. Cytotoxicity Evaluation

The cytotoxic effects of the tested materials on HGF‐1 cells were evaluated using the 3‐(4,5‐dimethylthiazol‐2‐yl)‐2,5‐diphenyl tetrazolium bromide (MTT) assay through both direct and indirect contact methods. For the direct cytotoxicity assay, 30,000 HGF‐1 cells were seeded onto the material discs and glass coverslips (control) placed in 96‐well tissue culture plates and incubated for 48 h under standard culture conditions. Subsequently, 10 μL of MTT solution was added to each well containing 100 μL of culture medium, followed by incubation for an additional 3 h. The reaction medium was then carefully removed, and 100 μL of isopropanol containing 0.1% hydrochloric acid was added to dissolve the formazan crystals. For the indirect cytotoxicity assay, each set material disc was immersed in complete DMEM and incubated at 37°C in a humidified atmosphere with 5% CO_2_ for 48 h to obtain material extracts. The resulting undiluted extract was defined as the 100% extract. Serial dilutions of the extract were then prepared using fresh culture medium to obtain concentrations of 50%, 25%, 12.5%, 6.25%, and 3.125% (v/v). HGF‐1 cells (30,000 cells per well) were exposed to each extract concentration for 48 h under standard culture conditions, after which the MTT assay was performed as described for the direct method. The optical density was measured at 540 nm using a multiwell microplate reader. Cell metabolic activity was expressed as a percentage relative to the control group. For each experimental condition, six independent samples were analyzed (*n* = 6), and all measurements were performed in triplicate. The results are presented as mean (±) standard deviation (SD).

### 2.4. Cell Morphology and Surface Interaction Analysis

Cell morphology, attachment, and surface interaction of HGF‐1 cells on all sample discs were evaluated by scanning electron microscopy (SEM). Initially, all samples were sterilized and positioned at the bottom of a 12‐well culture plate. Cells were then added to the wells at a density of 3 × 10^4^ cells per well for 48 h. Following incubation, the cells were rinsed three times with phosphate‐buffered saline (PBS) and subsequently fixed in a solution of 2.5% glutaraldehyde in a 1 mmol sodium cacodylate buffer at the pH of 7.4. To dehydrate the specimens, a series of ethanol concentrations (30%, 50%, 70%, 90%, and 100%) were employed. After drying, the specimens were coated with gold through sputter‐coating. Finally, the samples were examined using an XL30 SEM microscope (PHILIPS, Netherlands). For SEM evaluation, three representative discs per group were examined (*n* = 3) to qualitatively assess the cell morphology and surface interaction.

### 2.5. Cell Adhesion

HGF‐1 cells were enzymatically detached using 0.1% trypsin and seeded onto the prepared material discs placed in culture plates at an appropriate density. The cells were then incubated under standard culture conditions to allow attachment and spreading. After incubation for 24 and 48 h, nonadherent cells were gently removed by washing with PBS. The adherent cells were subsequently fixed with 4% paraformaldehyde for 20 min, followed by permeabilization. The actin cytoskeleton was stained with 70 nM fluorescent phalloidin (Sigma‐P1951) for 120 min, and cell nuclei were counterstained with 5 μg/mL 4′,6‐diamidino‐2‐phenylindole (DAPI; Sigma‐D9542) for 20 min. After staining, the samples were rinsed with PBS. Fluorescence images were acquired using a fluorescence microscope (Olympus BX51, Olympus Corp., Tokyo, Japan). Quantitative analysis of cell adhesion was performed by measuring fluorescence intensity using ImageJ software. Six independent samples per group were analyzed at each time point (24 and 48 h) (*n* = 6 per time point).

### 2.6. Statistical Analysis

The analysis of data was conducted using GraphPad Prism software, Version 8.0, with results presented as mean (±)SD. One‐ and two‐way ANOVA were performed, followed by a post hoc Tukey test for further analysis. *p* values, less than 0.05, were deemed statistically significant.

## 3. Results

### 3.1. Effects of GS’s Biocompatibility With Gingival Cells Compared to Glass Ionomers

**FIGURE 1:**
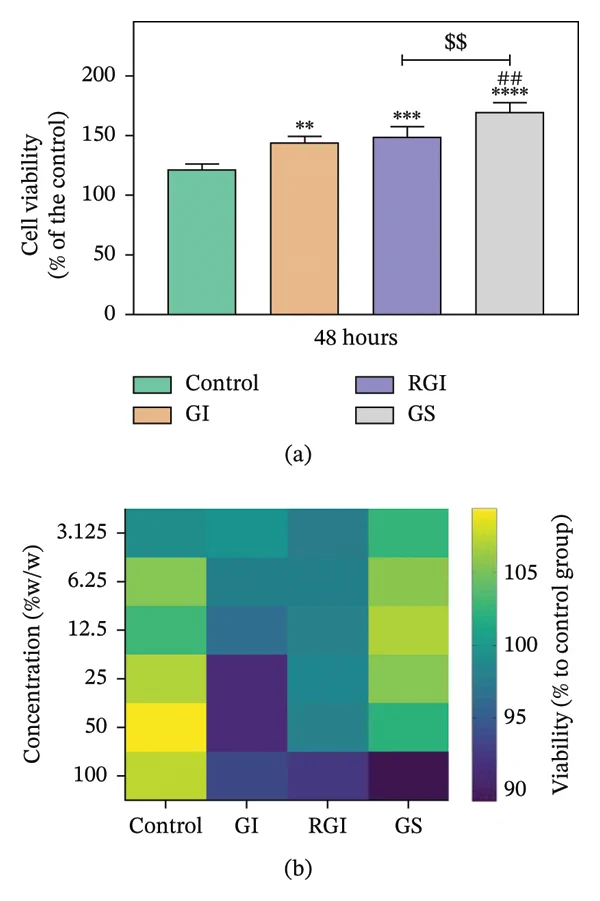
(a) MTT assay evaluating the metabolic activity and viability of HGF‐1 cells cultured on Geristore (GS), glass ionomer (GI) Fuji II, and resin‐modified glass ionomer (RGI) Fuji II LC after 48 h of incubation. Data are presented as mean ± SD (*n* = 6). ^∗^ *p* < 0.05 indicates a statistically significant difference compared with the glass coverslip control group. ^#^ *p* < 0.01 and ^$^ *p* < 0.0001 indicate the statistically significant differences between the GS group and the GI and RGI groups, respectively. (b) Heatmap showing the metabolic activity of HGF‐1 cells exposed to serial dilutions of material extracts after 48 h.

The results of MTT assay indicated the biocompatibility and nontoxicity of all samples compared to the control group (Figure 1). After 48 h of incubation, the HGF‐1 cell viability in GI, RGI, and GS groups remained significantly higher than that of the control group (*p* < 0.01, *p* < 0.001, and *p* < 0.0001, respectively). Moreover, the GS group exhibited a statistically significant increase in the metabolic activity of the living HGF‐1 cells compared to GI and RGI groups (*p* < 0.01 for both groups). Nevertheless, no significant difference was observed in cell metabolic activity between GI and RGI groups (Figure 1a).

Figure 1b illustrates the toxicity effects of various concentrations of samples after 48 h of incubation with HGFs, presented as a heat map. The plot indicates that increasing the concentration of the control sample corresponded to a heightened metabolic effect on the cells. In the GI group, no toxic effects were detected at concentrations below 25% W/W. However, concentrations at or above the mentioned level resulted in cytotoxic effects when compared to the control group. Notably, a toxicity effect was observed at a concentration of 100% W/W for both RGI and GS samples. Furthermore, concentrations below 50% W/W of the GS extract demonstrated an increase in cellular metabolic activity.

### 3.2. Comparative Ultrastructural Assessment of HGF‐1 Behavior on GS Versus Glass Ionomer Surfaces

**FIGURE 2:**
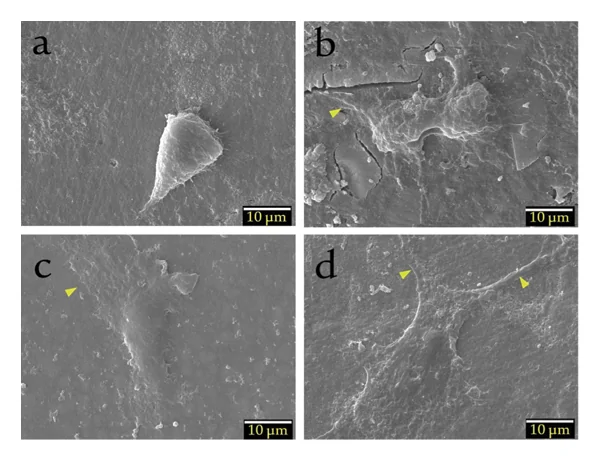
Representative scanning electron microscopy images showing the morphological characteristics and surface adhesion of HGF‐1 cells 48 h after incubation with (a) glass coverslip (control), (b) glass ionomer (GI) Fuji II, (c) resin‐modified glass ionomer (RGI) Fuji II LC, and (d) Geristore (GS). Arrowheads indicate cellular processes and filopodial extensions. Images were acquired at 4000x magnification. Scale bar = 10 μm.

Scanning electron microscopy revealed distinct differences in HGF‐1 cell morphology and surface interaction among the tested materials (Figure 2). In the control group (glass coverslips; Figure 2a), HGF‐1 cells exhibited limited spreading and failed to display the typical elongated fibroblastic morphology. The cells appeared relatively compact and lacked well‐defined filopodial extensions, suggesting weak surface interaction under these conditions. In contrast, HGF‐1 cells cultured on GI surfaces demonstrated improved attachment and spreading compared with the control group (Figure 2b). Cells on GI discs exhibited more pronounced cellular processes and partial elongation, indicating enhanced surface interaction. On RGI surfaces, fibroblasts showed further improvement in cell spreading, with a broader cell body and more evident filopodial extensions compared to GI (Figure 2c). The most pronounced morphological features were observed on GS surfaces (Figure 2d). HGF‐1 cells cultured on GS discs displayed an elongated fibroblastic morphology with extensive, well‐defined filopodia extending across the material surface. The cells appeared well‐spread and flattened, indicating strong adhesion and intimate interaction with the GS substrate.

### 3.3. Enhancing HGF‐1 Cell Attachment and Adhesion Surface Area on GS Material

**FIGURE 3:**
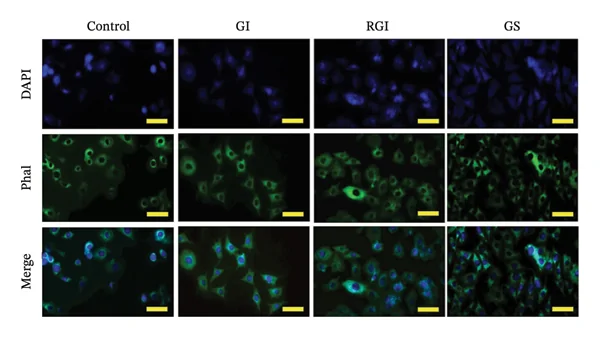
Phalloidin and DAPI staining reveal the attachment of HGF‐1 cells on control (coverslips), GI (Fuji II), RGI (Fuji II LC), and GS (Geristore) sample discs following a 24‐h incubation period. Images were captured using DAPI, phalloidin, and merge filters to display the nucleus, cell cytoplasm, and their combination, respectively. The scale bar indicates 20 μm.

**FIGURE 4:**
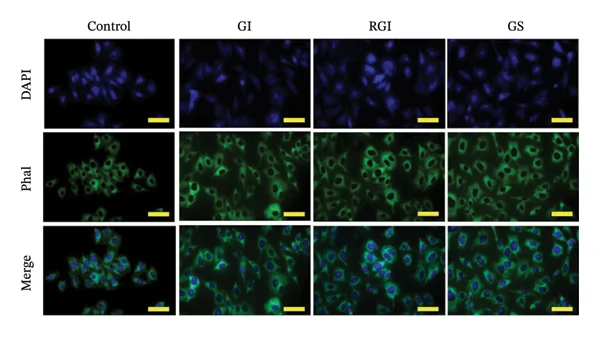
After a 48‐h incubation, phalloidin and DAPI staining also demonstrate the adhesion of HGF‐1 cells on the sample discs: control (coverslips), GI (Fuji II), RGI (Fuji II LC), and GS (Geristore). The images were taken with DAPI, phalloidin, and merge filters to illustrate the nucleus, cell cytoplasm, and their combination, with the scale bar representing 20 μm.

Figures 3 and 4 provide a detailed illustration of fluorescence staining performed on the cell cytoplasm using phalloidin, alongside the nuclear contrast facilitated by DAPI. The qualitative results obtained within the initial 24 h following incubation with the discs clearly indicate the adhesion of cells and the formation of cellular processes, specifically noted in the glass ionomers and GS samples. In contrast, the fibroblastic appearance within the control discs was observed to be barely discernible, highlighting a notable difference in cellular behavior between the experimental and control materials. Furthermore, it is evident that the GS discs exhibited a significantly greater density of cell adhesion when evaluated per unit area of the discs, as evidenced by both the count of viable nuclei and the prominent fibroblastic morphology displayed.

On the second day of observation, an increase in cell density was recorded across all sample groups. Additionally, the spindle‐shaped morphology of the HGF‐1 cells became apparent in all experimental samples. Nonetheless, it is important to note that the cell appearance in the control group samples remained relatively comparable to the other experimental groups. The cell fibers were specially more pronounced in the GS discs when compared to the other tested samples, suggesting a preferential adhesion on the mentioned specific materials.

**FIGURE 5:**
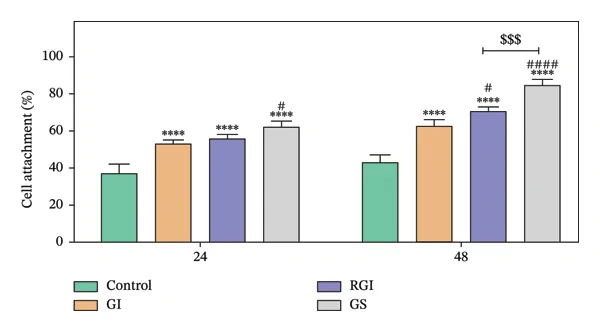
Quantitative analysis of HGF‐1 cell adhesion on geristore (GS), glass ionomer (GI) Fuji II, and resin‐modified glass ionomer (RGI) Fuji II LC after 24 and 48 h of incubation. Data are presented as mean ± SD (*n* = 6). ^∗^ *p* < 0.05 indicates a statistically significant difference compared with the glass coverslip control group. ^#^ *p* < 0.001 and ^$^ *p* < 0.0001 indicate the statistically significant differences between the GS group and the GI and RGI groups, respectively.

The findings related to the quantification of cell adhesion on the various samples are illustrated in Figure 5. On the first day of analysis, a statistically significant difference in cell attachment was observed on the GI, RGI, and GS discs when compared to the control group, with a *p* value of less than 0.0001 applicable to all experimental groups. Notably, this significant difference in cell adhesion was sustained and evident on the second day of evaluation, reinforcing the reliability of the results. Moreover, the comparison of cell adhesion between the GS samples and the GI and RGI groups reached its peak on the second day (*p* < 0.001 and *p* < 0.0001, respectively). Additionally, a significant increase in the adhesion of HGF‐1 cells was noted on this day specifically for the RGI group when compared to the GI group (*p* < 0.05). These results collectively emphasize the differential adhesive properties of the materials studied and their influence on cellular behavior over time. The analysis of the interaction effect of time within the samples of each group is detailed in Figure 3.

## 4. Discussion

With advancements in dental materials, particularly glass ionomers and GS, there is a growing interest in their applications for clinical procedures; e.g., CL and DME [7]. Not only do these materials serve as effective restoratives, but they play a crucial role in the enhancement of periodontal health and tissue regeneration [10]. Biocompatibility is a crucial issue to be deliberated, as leached components from the materials can significantly impact the biological responses of surrounding tissues [24]. To the best of our knowledge, this is the first study to investigate the cellular response to materials used in DME through in vitro analysis, providing novel insights into the biological interactions at the cellular level.

According to the ISO 10993‐5 standard, in vitro cytotoxicity testing is widely used as a primary screening tool for evaluating the biocompatibility of medical and dental materials, with cell viability values above approximately 70% generally considered indicative of noncytotoxic behavior [25]. In the present study, all tested materials demonstrated cell viability levels exceeding this commonly accepted threshold, confirming their basic biocompatibility. However, it is important to note that ISO 10993‐5 allows methodological flexibility, and variations under experimental conditions may influence absolute viability values, highlighting the importance of contextual interpretation rather than reliance on a single cutoff value [25, 26].

Beyond meeting minimum biocompatibility thresholds, materials intended for subgingival applications, e.g., DME, should support adequate cell adhesion and favorable cellular behavior. Enhanced gingival fibroblast attachment and metabolic activity, as observed for GS in the present study, may reflect a more biologically favorable surface for soft tissue interaction. Previous studies have demonstrated that substrate properties, including surface characteristics and stiffness, significantly influence gingival fibroblast viability, adhesion, and resistance to cytotoxic stress, emphasizing the clinical relevance of evaluating cell–material interactions beyond standard tissue culture plastic controls [27]. From a clinical perspective, increased fibroblast adhesion and sustained metabolic activity may contribute to improved soft tissue integration and periodontal stability when restorative materials are placed in close proximity to gingival tissues [28]. Therefore, while all evaluated materials fulfilled established biocompatibility criteria, the superior cellular response observed on GS may offer additional biological advantages in DME procedures, where long‐term soft tissue compatibility is critical for restorative success.

In the present study, the biocompatibility of GS was compared with conventional and RGI cements using complementary viability and cell–material interaction analyses. All tested materials demonstrated noncytotoxic behavior toward HGF‐1 gingival fibroblasts after 48 h of incubation, while GS exhibited significantly higher metabolic activity and improved fibroblast adhesion compared with GI and RGI [29]. These findings suggest that, beyond basic biological safety, GS provided a more favorable surface for gingival fibroblast interaction. The HGF‐1 cell line was selected as a clinically relevant in vitro model, as gingival fibroblasts play a central role in periodontal tissue maintenance and healing. Consistent with this, fluorescence‐based adhesion analysis further confirmed a significantly greater density of adherent cells on GS surfaces compared with the control group, supporting its enhanced biological compatibility. Although the present study did not directly replicate a clinical DME model, the observed cellular responses offer meaningful insight into the biocompatibility and cell–material interactions of restorative materials under conditions relevant to subgingival applications and may inform future studies aimed at optimizing materials for DME.

Dental caries and structural tooth defects remain among the leading causes of tooth loss, and restorative management of lesions extending into the biological width or subcrestal regions continues to present clinical challenges [13, 30, 31]. DME, also referred to as coronal margin relocation, has been proposed as a conservative approach to reposition deep margins coronally, thereby improving marginal sealing and facilitating restorative procedures while preserving periodontal health [6].

Glass ionomer–based materials are widely used in restorative dentistry due to their favorable adhesion to dental tissues, fluoride release, and generally acceptable biocompatibility profiles [32]. Conventional glass ionomers have been shown to exhibit low cytotoxicity and satisfactory adaptation to both soft and hard tissues, supporting their use in procedures where maintenance of the biologic width is critical RGIs offer additional mechanical strength and improved handling characteristics, including reliable adhesion under moist conditions, which has contributed to their use in a variety of restorative indications [33–35]. These properties have positioned glass ionomer materials as commonly employed options in DME procedures; however, continued evaluation of their biological performance remains necessary to optimize material selection for subgingival applications [36].

The cytotoxicity assessments indicated that both GI and RGI discs exhibited noncytotoxic behavior toward HGF‐1 cells, although previous studies have reported variable cytotoxic responses for RGIs [37]. Such discrepancies across the literature are often attributed to methodological differences, including the use of fresh versus set materials, direct contact versus extract‐based assays, and variations in extraction conditions [9, 13]. In the present study, evaluating cytotoxicity directly on material discs was intended to better approximate clinical conditions, as restorative materials in vivo remain in continuous contact with surrounding tissues rather than isolated extracts. Extract‐based testing has shown that cellular metabolic responses to RGI may vary with concentration and exposure conditions [14]; however, in our experiments, no statistically significant differences were observed across the tested extract concentrations. This finding further supports the notion that extract‐based assays may not always accurately reflect clinical behavior, particularly given that prolonged immersion can lead to progressive washout of leachable components [11]. In the oral and periapical environments, restorative materials are constantly exposed to blood and tissue fluids, which may modulate the release and dilution of potentially cytotoxic substances over time [29]. Consistent with this concept, our results suggested that increased washout was associated with reduced cytotoxic effects for GI and RGI, while GS demonstrated a trend toward enhanced biocompatibility following washout. These observations highlight the importance of considering both testing methodology and dynamic clinical conditions when interpreting cytotoxicity data for materials intended for subgingival applications.

The favorable cellular responses observed on GS surfaces suggested its potential to support periodontal tissue compatibility in subgingival restorative applications. In the present study, GS demonstrated significantly higher cellular metabolic activity and more pronounced fibroblast adhesion compared to GI and RGI, predominantly at later time points. These effects may be attributed to the surface characteristics of GS, including its granular topography with smooth edges and grooves, which may provide a more favorable scaffold for cell attachment [9]. In addition, GS has been reported to release fewer potentially cytotoxic substances into the surrounding medium, further supporting its suitability for applications, e.g., DME [38].

Microscopic evaluation by SEM revealed extensive cell spreading and well‐developed filopodia on GS discs, indicating stable cell–material interaction, whereas more limited spreading was observed on GI and RGI surfaces. These observations are consistent with previous reports demonstrating preferential gingival fibroblast attachment to GS compared to other restorative materials [19]. It has been suggested that cell attachment and morphology on a material surface may serve as sensitive indicators of biological compatibility [39]. The enhanced surface‐associated cellular behavior observed on GS may be related to its material composition and bioactive properties, as surface‐mediated ionic interactions have been shown to influence fibroblast attachment and activity [40]. These findings support the notion that GS could provide a favorable surface for gingival fibroblast interaction, which may be beneficial for maintaining soft tissue stability during restorative procedures involving subgingival margins.

The formation of filopodia and the characteristic fibroblastic morphology observed on GS surfaces indicated a favorable substrate for gingival fibroblast attachment, which is crucial for maintaining periodontal tissue stability during restorative procedures. This is particularly relevant in DME, where the goal is to elevate the restorative margin while preserving the integrity of the surrounding periodontal structures [41]. The ability of GS to promote stable fibroblast adhesion and favorable cellular morphology may contribute to the preservation of a healthy biologic width [42]. This is critical, as the maintenance of the biological width is essential for preventing periodontal complications, e.g., tissue inflammation and attachment loss following restorative interventions [32]. The study by Gupta et al. further supports this concept by demonstrating that the extensive cellular morphology observed on GS surfaces is associated with enhanced periodontal healing responses [10]. As a hydrophilic, polyacid‐modified composite resin, GS incorporates fluoride‐releasing glass particles within an organic polymerizable matrix activated by a photo‐initiator [7]. These material characteristics, together with its documented biocompatibility, may trigger its desirable interaction with periodontal tissues [32, 43]. Histological analyses have also demonstrated robust cell attachment and normal tissue morphology on GS surfaces [44]. Nevertheless, direct comparative studies evaluating the biocompatibility of GS relative to other materials specifically used for DME remain limited.

Previous fluorescence‐based studies have reported higher fibroblast adhesion to GS compared to other restorative materials, including gingival and periodontal ligament fibroblasts [45]. The fluorescence quantification results of the present study are consistent with these findings, demonstrating significantly greater cell attachment on GS surfaces. Notably, this study is among the first to apply dual fluorescence staining to concurrently assess nuclear integrity and cytoskeletal organization in the context of materials relevant to DME [38]. The combined use of DAPI and phalloidin enabled a more detailed evaluation of cell attachment and cytoskeletal organization on restorative material surfaces, providing additional insight into cell–material interactions under conditions relevant to subgingival applications. Consistent with the quantitative data, microscopic observations revealed enhanced and time‐dependent fibroblast adhesion on GS discs compared to other tested materials. This behavior may be partially attributed to the surface topography of GS, as its granular structure with smooth edges may have suggested to provide a favorable scaffold for cell attachment [10]. In addition, GS has been reported to release fewer potentially cytotoxic components into the surrounding medium, which could further support sustained cell attachment over time [19]. These findings reinforce the favorable biological profile of GS and highlight the value of dual fluorescence staining for characterizing cellular responses to restorative materials. To the best of our knowledge, the application of such a combined staining approach to evaluate cell behavior in the context of DME has not been previously reported.

Primary cell cultures and established cell lines are commonly employed to evaluate the biocompatibility and biological behavior of dental materials, as cellular responses may vary depending on exposure conditions and duration [2, 24]. In the present study, the biocompatibility of materials used for DME was assessed via the comparison of GS with conventional GIs and RMGIs using complementary viability, adhesion, and morphological analyses at two time points. The use of HGF‐1 gingival fibroblasts as an experimental model is chiefly relevant, as these cells play a central role in maintaining periodontal tissue integrity and mediating soft tissue responses to restorative materials [46, 47]. The observed cellular responses indicate that GS provides a biologically encouraging surface for gingival fibroblast interaction compared to GI and RGI. From a clinical perspective, the application of DME using materials with enhanced soft tissue compatibility may support a more conservative restorative approach by facilitating coronal relocation of deep margins while preserving periodontal attachment and reducing the risk of microleakage [17]. These findings suggested that GS could represent a promising material for subgingival restorative procedures; however, further in vivo and long‐term clinical studies are required to validate its performance under clinical conditions.

Although the experimental techniques employed in this study, including MTT assays, SEM analysis, and fluorescence staining, have been widely used in the evaluation of dental biomaterials, the present work differs from previous studies in its specific focus on GS within the context of DME. By directly comparing GS with conventional and RGIs using a gingival fibroblast model, this study provides material‐specific insights into cell–surface interactions relevant to subgingival restorative procedures. Future investigations should further elucidate how materials used for DME modulate cellular responses, thereby establishing a stronger biological framework for their clinical application. Although cytotoxicity assessment is an essential component of biocompatibility evaluation, its individuality is insufficient to predict clinical performance, and additional parameters related to cell–material interaction and tissue response should be considered. The present study is limited by its in vitro design, which cannot fully replicate the complexity of clinical conditions. Therefore, further long‐term and in vivo studies are warranted to validate these findings and to better define the clinical relevance of materials used in DME.

## 5. Conclusions

Given the considerable challenges of dental defects present in restoration(s) and margin management, it is crucial to explore restorative materials with appropriate biocompatibility properties to improve the biological response(s) of periodontal tissues. The current study evaluated the biocompatibility of GS in comparison with Fuji II and Fuji II LC glass ionomers, focusing on their effects on performance and success in DME. The findings indicated that GS demonstrated superior biocompatibility, significantly enhancing the adhesion of HGF cells. The surface properties of GS seemed to promote the optimal conditions for cell attachment compared to conventional glass ionomers. Furthermore, GS exhibited a greater rate of cell adhesion at two distinct time intervals, emphasizing its potential to facilitate soft tissue healing and generate favorable biological response(s). Additionally, SEM analysis illustrated effective cell spreading on GS surfaces, preserving natural cell morphology, whereas cell adhesion on conventional glass ionomers seemed more constrained. The obtained results can position GS as a promising candidate for clinical applications that require biological compatibility and reparative properties, particularly in DME. While further research is warranted to investigate the long‐term in vivo effects of GS, its anticipated clinical adoption appears encouraging.

## Author Contributions

Conceptualization: Mahsa Radafshar.

Formal analyses: Mahsa Radafshar and Ardavan Parhizkar.

analysis: Mahsa Radafshar and Ardavan Parhizkar.

Investigation: Mahsa Radafshar and Ardavan Parhizkar.

Methodology: Mahsa Radafshar and Ardavan Parhizkar.

Project administration: Mahsa Radafshar and Ardavan Parhizkar.

Supervision: Ardavan Parhizkar.

Validation: Mahsa Radafshar and Ardavan Parhizkar.

Visualization: Mahsa Radafshar and Ardavan Parhizkar.

Writing–original draft: Mahsa Radafshar.

Writing–review and editing: Mahsa Radafshar and Ardavan Parhizkar.

## Funding

The authors affirm that they have no financial affiliation or involvement with any commercial organization with direct/indirect financial interest in the subject or materials discussed in this manuscript, nor have any such arrangements. This study received no funding.

## Conflicts of Interest

The authors declare no conflicts of interest.

## Data Availability

The data used to support the findings of this study are available upon reasonable request from the corresponding author.
